# Mental Health Treatment of Individuals Seeking Holy Water Treatment in Ethiopia

**DOI:** 10.1001/jamanetworkopen.2025.36558

**Published:** 2025-10-08

**Authors:** Solomon Moges Demeke, Kindie Mekuria Tegegne, Atitegeb Abera Kidie, Fentaw Girmaw Denberu

**Affiliations:** 1 Department of Psychiatry, College of Health Science, Woldia University, Woldia, Ethiopia; 2 Department of Public Health, College of Health Science, Woldia University, Woldia, Ethiopia; 3 Department of Pharmacy, Education and Service Directorate of Pharmacy, College of Health Sciences, Woldia University, Woldia, Ethiopia

## Abstract

**Importance:**

A treatment gap reflects the disparity between those with mental health disorders and those receiving care, especially in low- and middle-income countries, where many remain untreated.

**Objective:**

To assess the mental health treatment gap among individuals seeking holy water healing in Ethiopia and identify associated factors.

**Design, Setting, and Participants:**

This mixed-methods cross-sectional study was conducted from March 1 to August 31, 2024, in North Wollo, Ethiopia. Individuals with symptoms of mental illness were surveyed using interviewer-administered questionnaires. Data were analyzed from October to December 2024.

**Exposures:**

Sociodemographic, behavioral, psychosocial, and clinical factors were assessed using culturally adapted, structured questionnaires based on existing literature.

**Main Outcomes and Measures:**

The General Help-Seeking Questionnaire was used to assess participants’ use of professional mental health services. Qualitative data from focus group discussions and key informant interviews were thematically analyzed and triangulated with quantitative findings.

**Results:**

A total of 393 participants (mean [SD] age, 30.06 [9.87] years; 201 [51.1%] female) responded to the survey, including 144 (36.6%) younger than 25 years, 375 (95.4%) Orthodox Christians, and 270 (68.7%) residing in rural areas. Traditional healers were consulted by 242 participants (61.6%), whereas only 70 (17.8%) accessed professional mental health care. Fear of being seen as bad parents was cited by 377 (95.9%) as a key barrier. Overall, 323 participants (82.2%; 95% CI, 78.1%-86.0%) experienced a treatment gap. Factors associated with the gap included low perceived need for care (adjusted odds ratio [AOR], 2.49; 95% CI, 1.09-5.71), male gender (AOR, 2.32; 95% CI, 1.20-4.48), higher educational level (AOR, 4.34; 95% CI, 1.79-10.53), low income (AOR, 2.41, 95% CI, 1.72-4.14), not knowing where to go (AOR, 1.97; 95% CI, 1.21-4.53), denial (AOR, 3.21; 95% CI, 1.24-6.43), and internalized stigma (AOR, 2.76; 95% CI, 1.32-5.80). Qualitative results reinforced these and highlighted cultural beliefs, weak social support, and limited institutional focus as additional barriers.

**Conclusions and Relevance:**

In this cross-sectional study, only 1 in 6 individuals with mental health symptoms sought professional care. The treatment gap was associated with sociocultural, economic, and systemic challenges. Addressing these factors requires culturally sensitive interventions, public education, improved access, and stronger mental health systems.

## Key Points

**Question:**

Is there a mental health treatment gap among individuals seeking holy water treatment in Ethiopia?

**Findings:**

In this cross-sectional study of 393 participants with mental illness symptoms, 323 (82.2%) had a treatment gap. Factors associated with this gap included lack of perceived need, male gender, higher educational level, low income, uncertainty about help sources, denial, and high internalized stigma.

**Meaning:**

This study’s findings suggest that addressing the large mental health treatment gap requires culturally sensitive, accessible, and stigma-reducing interventions tailored to identify barriers.

## Teaser

This cross-sectional study explores whether there is a mental health treatment gap among individuals seeking holy water treatment in Ethiopia and how cultural beliefs, stigma, and socioeconomic barriers are associated with help-seeking behaviors.

## Introduction

Mental illnesses are the leading cause of disability worldwide, disproportionately affecting low- to middle-income countries (LMICs).^1,2,3^ The 2021 Global Burden of Disease Study reported that 1.1 billion people live with mental disorders globally, with sub-Saharan Africa bearing 22% of this burden despite comprising only 14% of the world’s population.^4^ In Ethiopia, recent estimates suggest that 20% to 25% of adults experience mental health conditions, yet treatment coverage remains below 10%.^5^ Significant numbers of people living with mental illness do not receive treatment or do not seek help from practitioners of formal modern treatment.^6^ Notably, two-thirds (66.7%) of those affected live in LMICs, where mental health resources are critically limited.^4^ Mental disorders contribute significantly to disability, accounting for 22.7% to 37% of total years lived with disability globally. Despite affecting up to 30% of the population annually, approximately two-thirds of those needing treatment receive no intervention.^7,8,9^

The treatment gap is defined as the difference between the prevalence of mental disorders and the proportion receiving appropriate care. It is a key indicator of unmet mental health needs.^10,11,12^ This gap is particularly pronounced in LMICs, where 76% to 84% of individuals with severe mental illnesses remain untreated annually^9^ compared with 35% to 50% in high-income countries.^13^ In Europe, although 38.2% of the population has mental illnesses, less than one-third receive professional care. Factors contributing to the treatment gap include stigma, low perceived need for treatment, limited mental health literacy, and systemic barriers to care.^14,15,16,17,18^ The treatment gap in LMICs is concerning.^9,19^ These countries account for 85% of the global population and bear nearly three-quarters of the global mental health burden,^20,21^ yet they possess less than 20% of global mental health resources.^22^ In low-income countries, access to treatment often falls below 10%, with nearly 90% of mental disorder cases untreated.^13,19,23^ Untreated mental illnesses have far-reaching consequences, including increased criminal activity, reduced employment, strained relationships, homelessness, and premature mortality.^24,25,26,27^ Individuals with schizophrenia and major depression face a 40% to 60% higher risk of premature death. Untreated mental disorders also reduce productivity and increase health care and welfare costs.^28,29,30^

Cultural and religious beliefs substantially influence mental health treatment-seeking behaviors in LMICs. Magicoreligious beliefs often lead individuals to seek help from traditional or spiritual healers instead of professional psychiatric services.^31^ In South Africa, 61% of individuals with mental illnesses consult faith healers.^32^ In Ethiopia, many rely on traditional remedies, such as holy water and spirit mediums, often associated with Ethiopian Orthodox Christianity. Despite widespread use of spiritual healing, those seeking holy water treatment rarely receive referrals to psychiatric services, leaving their needs unmet.^33,34,35^ This study aims to assess the mental health treatment gap and its associated factors among individuals seeking holy water healing in North Wollo, Ethiopia, and to explore how cultural beliefs, stigma, and socioeconomic barriers influence their help-seeking behaviors. By integrating quantitative and qualitative methods, this study aims to bridge the gap between spiritual and biomedical mental health care in a region where holy water is a primary intervention.

## Methods

### Design, Setting, and Participants

This cross-sectional study used a mixed-methods design to investigate the mental health treatment gap at holy water sites in North Wollo Zone, Amhara, Ethiopia, from March 1 to August 31, 2024. The zone, located in northeastern Ethiopia, has a specialty hospital, 6 hospitals, and 19 health facilities. The study focused on 6 holy water sites (eFigure in Supplement 1). A total of 412 participants with mental health symptoms were selected using simple random sampling. Investigators recorded participants’ self-reported religion, race, and ethnicity. These data are essential because these factors are strongly tied to cultural beliefs, stigma, health care access, and socioeconomic status all fundamental determinants of mental health treatment–seeking behavior. A few participants did not report this information and were considered nonrespondents. Consequently, the final analyzed sample size was 393 participants. Lists of individuals using holy water at each selected church were obtained from registration books, where attendants are recorded on entry. After these lists were compiled, participants were selected with the lottery method, using simple random sampling. Eligibility criteria included age older than 18 years, presence of mental illness symptoms, and oral informed consent. Those with cognitive impairment or physical incapacitation were excluded. We trained data collectors and supervisors for 2 days. Data collection involved semistructured questionnaires and qualitative interviews conducted by 4 bachelor’s degree holders in psychiatry, supervised by a master’s degree holder specializing in mental health. Quantitative data covered sociodemographic, clinical, and psychosocial factors, assessed through validated tools such as the World Health Organization Disability Assessment Schedule; Oslo Social Support Scale; Barriers to Access to Care Evaluation; Alcohol, Smoking, and Substance Involvement Screening Test; Cut down, Annoyed, Guilty, Eye-opener Adapted to Include Drugs; and General Help-Seeking Questionnaire. The qualitative component included focus group discussions (FGDs) and key informant interviews (KIIs), exploring experiences with mental health services and help-seeking behaviors. The interview guide included questions about participants’ characteristics, their perceptions of mental health services, patterns of help-seeking behavior for mental health problems, and potential barriers preventing psychiatric patients from accessing professional care. Data collection continued until theme saturation, with all interviews audio-recorded with consent. Procedures for this cross-sectional study were approved by the Woldia University institutional review board. Oral informed consent was obtained from all study participants. Confidentiality was maintained throughout the study, and data collectors received ethical training to ensure adherence to ethical standards. The study adhered to the Strengthening the Reporting of Observational Studies in Epidemiology (STROBE) guidelines for observational research, ensuring comprehensive and ethical data reporting. Our approach to qualitative analysis and study design was guided by relevant portions of the Consolidated Criteria for Reporting Qualitative Research (COREQ) reporting guideline.

### Statistical Analysis

SPSS, version 25 (IBM Inc) was used for quantitative analysis. Descriptive statistics summarized the data. Bivariate logistic regression identified candidate variables with *P* < .25, which were then entered into a multivariable logistic regression model to identify factors associated with the treatment gap. Associations were reported using adjusted odds ratios (AORs) with 95% CIs, and statistical significance was set at a 2-sided *P* < .05. Qualitative data were transcribed, translated, and thematically analyzed using Open Code, version 3.4 (UMEU Centre at the Department of Epidemiology and Global Health, Umeå University).^36^ Data were analyzed from October to December 2024.

## Results

### Sociodemographic Characteristics 

This study included 393 adult participants (mean [SD] age, 30.06 [9.87] years; 201 [51.1%] female and 192 [48.9%] male). A total of 144 participants (36.6%) were younger than 25 years, 375 (95.4%) were Orthodox Christians, 136 (34.6%) were single, and 270 (68.7%) lived in rural areas. Moreover, 331 (84.2%) had a personal monthly income of 3729.97 Ethiopian Birr (ETB) or less (US $1.00 = 57.829 ETB), and 363 (92.4%) had a family monthly income of 3729.97 ETB or less (Table 1).

**Table 1.** Characteristics of Study Participants

Characteristic	No. (%) of participants (N = 393)
Gender	
Male	192 (48.9)
Female	201 (51.1)
Age, y	
<25	144 (36.6)
25-34	132 (33.6)
35-44	78 (19.8)
≥45	39 (9.9)
Marital status	
Married	132 (33.6)
Single	136 (34.6)
Divorced	92 (23.4)
Widowed	33 (8.4)
Religion	
Orthodox Christian	375 (95.4)
Muslim	18 (4.6)
Residence	
Urban	123 (31.3)
Rural	270 (68.7)
Educational status	
Unable to read and write	180 (45.8)
Primary level	78 (19.8)
Secondary level	53 (13.5)
College and above	82 (20.9)
Occupational status	
Farmer	185 (47.1)
Merchant	57 (14.5)
Employed (private and government)	93 (23.7)
Jobless	28 (7.1)
Others^a^	30 (7.6)
Living status	
Alone	122 (31.0)
Partner	120 (30.5)
Family	101 (25.7)
Relatives	26 (6.6)
Friends	24 (6.1)
Mean monthly personal income, ETB	
≤3729.97	331 (84.2)
>3729.97	62 (15.8)
Mean monthly relative income, ETB	
≤ 3729.97	363 (92.4)
> 3729.97	30 (7.6)
Perceived treatment	
No	328 (83.5)
Yes	65 (16.5)
Presence of major medical illnesses	
No	361 (91.9)
Yes	32 (8.1)
Social support	
Poor	178 (45.3)
Medium	118 (30.0)
Strong	97 (24.7)
Stigma	
Low	150 (38.2)
High	243 (61.8)
Lifetime substance use	
No	180 (45.8)
Yes	213 (54.2)
Current substance use	
No	316 (80.4)
Yes	77 (19.6)
Substance abuse	
No	74 (18.8)
Yes	3 (0.8)
WHODAS score	
Below the mean	201 (51.1)
Higher the mean	192 (48.9)
Unsure where to go	
No	277 (70.5)
Yes	116 (29.5)
Thinking has no problem	
No	224 (57.0)
Yes	169 (43.0)
Professional help will not work	
No	234 (59.5)
Yes	159 (40.5)
Concern what friends think	
No	247 (62.8)
Yes	146 (37.2)
Wanting to solve problem on my own	
No	219 (55.7)
Yes	174 (44.3)

Abbreviations: ETB, Ethiopian Birr; WHODAS, World Health Organization Disability Assessment Schedule.

^a^ Student, jobless, or daily labor.

### Treatment-Seeking for Mental Illnesses 

Of the 180 participants (45.8%) unable to read or write, 18 (10.0%) reported seeking mental health care. Of the 82 participants (20.9%) who had a college education or higher, 27 (32.9%) reported seeking mental health care. Among individuals with low income (mean monthly person income ≤3729.97 ETB), 200 (60.4%) relied on traditional healers, whereas 29 (46.8%) of participants with higher income sought professional care (Table 2).

**Table 2.** Treatment-Seeking Behavior for Mental Illnesses Among Participants Who Sought Spiritual Healing Through Holy Water at Selected Sites in North Wollo Zone, Amhara, Ethiopia, 2024

Characteristic	Treatment sought for mental illnesses, No. (%) (N = 393)					
	Total	Professionals		Nonprofessionals		
		Mental health care	General health care	Traditional healers and others	Any non–health care practitioner	Other sources
Gender						
Male	192 (48.9)	45 (23.4)	37 (19.3)	124 (64.6)	89 (46.4)	10 (5.2)
Female	201 (51.1)	25 (12.4)	29 (14.4)	118 (58.7)	83 (41.3)	3 (1.5)
Age, y						
<25	144 (36.6)	24 (16.7)	19 (13.2)	88 (61.1)	58 (40.3)	5 (3.5)
25-34	132 (33.6)	20 (15.2)	24 (18.2)	82 (62.1)	58 (43.9)	3 (2.3)
35-44	78 (19.8)	19 (24.4)	16 (20.5)	52 (66.7)	38 (48.7)	3 (3.9)
≥45	39 (9.9)	7 (17.9)	7 (17.9)	20 (51.3)	18 (46.2)	2 (5.1)
Marital status						
Married	132 (33.6)	14 (10.6)	23 (17.4)	79 (59.8)	53 (40.2)	3 (2.3)
Single	136 (34.6)	32 (23.5)	20 (14.7)	88 (64.7)	63 (46.3)	6 (4.4)
Divorced	92 (23.4)	19 (20.7)	19 (20.7)	60 (65.2)	41 (44.6)	4 (4.4)
Widowed	33 (8.4)	5 (15.2)	4 (12.1)	15 (45.5)	15 (45.5)	0
Religion						
Orthodox Christian	375 (95.4)	69 (18.4)	64 (17.1)	232 (61.9)	163 (43.5)	12 (3.2)
Muslim	18 (4.6)	1 (5.6)	2 (11.1)	10 (55.6)	9 (50.0)	1 (5.6)
Residence						
Urban	123 (31.3)	29 (23.8)	21 (17.1)	81 (65.9)	58 (47.2)	3 (2.4)
Rural	270 (68.7)	41 (15.2)	45 (16.7)	161 (59.6)	114 (42.2)	10 (3.7)
Educational status						
Unable to read and write	180 (45.8)	18 (10.0)	31 (17.2)	123 (68.3)	86 (47.8)	6 (3.3)
Primary level	78 (19.8)	15 (19.2)	16 (20.5)	45 (57.7)	32 (41.0)	3 (3.8)
Secondary level	53 (13.5)	10 (18.9)	6 (11.3)	23 (43.4)	23 (43.4)	2 (3.8)
College and above	82 (20.9)	27 (32.9)	13 (15.9)	51 (62.2)	31 (37.8)	2 (2.4)
Occupational status						
Farmer	185 (47.1)	37 (20.0)	28 (15.1)	111 (60.0)	79 (42.7)	6 (3.2)
Merchant	57 (14.5)	9 (15.8)	8 (14.0)	34 (59.7)	30 (52.6)	1 (1.8)
Employed	93 (23.7)	12 (12.9)	20 (21.5)	58 (62.4)	40 (43.0)	4 (4.3)
Jobless	28 (7.1)	5 (17.6)	3 (10.7)	20 (71.4)	10 (35.7)	0
Others^a^	30 (7.6)	7 (23.3)	7 (23.3)	19 (63.3)	13 (43.3)	2 (6.7)
Living status						
Alone	122 (31.0)	23 (18.9)	24 (19.7)	62 (50.8)	54 (44.3)	1 (0.8)
Partner	120 (30.5)	26 (21.7)	16 (13.3)	78 (65.0)	54 (45.0)	3 (2.5)
Family	101 (25.7)	15 (14.9)	16 (15.8)	64 (63.4)	44 (43.6)	7 (6.9)
Relatives	26 (6.6)	3 (11.5)	8 (30.8)	20 (76.9)	10 (38.5)	1 (3.8)
Friends	24 (6.1)	3 (12.5)	2 (8.3)	18 (75.0)	10 (41.7)	1 (4.7)
Mean monthly personal income, ETB						
≤3729.97	331 (84.2)	41 (12.4)	60 (18.1)	200 (60.4)	145 (43.8)	11 (3.3)
>3729.97	62 (15.8)	29 (46.8)	6 (9.7)	42 (67.7)	27 (43.5)	2 (3.2)
Mean monthly relative income, ETB						
≤3729.97	363 (92.4)	53 (14.6)	63 (17.4)	222 (61.2)	156 (42.9)	11 (3.0)
>3729.97	30 (7.6)	17 (56.7)	3 (10.0)	20 (66.7)	16 (53.3)	2 (6.7)
Perceived treatment						
No	328 (83.5)	52 (15.9)	39 (11.9)	197 (60.1)	130 (39.6)	11 (3.4)
Yes	65 (16.5)	18 (27.7)	27 (41.5)	45 (69.2)	42 (64.6)	2 (3.1)
Major medical illnesses						
No	361 (91.9)	63 (17.5)	65 (18.0)	222 (61.5)	160 (44.3)	13 (3.6)
Yes	32 (8.1)	7 (21.9)	1 (3.13)	20 (62.5)	12 (37.5)	0
Social support						
Poor	178 (45.3)	33 (18.5)	36 (20.2)	103 (57.9)	73 (41.0)	7 (3.9)
Medium	118 (30.0)	29 (24.6)	21 (17.8)	85 (72.0)	54 (45.8)	1 (0.9)
Strong	97 (24.7)	8 (8.3)	9 (9.3)	54 (55.7)	45 (46.4)	5 (5.2)
Stigma						
Low	150 (38.2)	16 (10.7)	32 (21.3)	89 (59.3)	69 (46.0)	3 (2.0)
High	243 (61.8)	54 (22.2)	34 (13.9)	153 (62.9)	103 (42.4)	10 (4.1)
Lifetime substance use						
No	180 (45.8)	34 (18.9)	33 (18.3)	120 (66.7)	77 (42.8)	7 (3.9)
Yes	213 (54.2)	36 (16.9)	33 (15.5)	122 (57.3)	95 (44.6)	6 (2.8)
Current substance use						
No	316 (80.4)	56 (17.7)	49 (15.5)	198 (62.7)	124 (39.2)	10 (3.2)
Yes	77 (19.6)	14 (18.2)	17 (22.1)	44 (57.1)	48 (62.3)	3 (3.9)
Substance abuse						
No	74 (18.8)	11 (14.9)	13 (17.6)	44 (59.5)	47 (63.5)	1 (1.4)
Yes	3 (0.8)	1 (33.3)	2 (66.7)	2 (66.7)	2 (66.7)	0
WHODAS score						
Below the mean	201 (51.1)	39 (19.4)	27 (13.4)	118 (58.7)	92 (45.8)	8 (3.9)
Higher the mean	192 (48.9)	31 (16.2)	39 (20.3)	124 (64.6)	80 (41.7)	5 (2.6)
Unsure where to go						
No	277 (70.5)	60 (21.7)	43 (15.5)	166 (59.9)	124 (44.8)	10 (3.6)
Yes	116 (29.5)	10 (8.6)	23 (19.8)	76 (65.5)	48 (41.4)	3 (2.6)
Thinking has no problem						
No	224 (57%)	49 (21.9)	42 (18.8)	143 (63.8)	92 (41.1)	9 (4.0)
Yes	169 (43%)	21 (12.4)	24 (14.2)	99 (58.6)	80 (47.3)	4 (2.4)
Professional help will not work						
No	234 (59.5)	50 (21.4)	41 (17.5)	148 (63.3)	101 (43.2)	10 (4.3)
Yes	159 (40.5)	20 (12.6)	25 (15.7)	94 (59.1)	71 (44.7)	3 (1.9)
Concern what friends think						
No	247 (62.8)	52 (21.1)	45 (18.2)	159 (64.4)	107 (43.3)	8 (3.2)
Yes	146 (37.2)	18 (12.3)	21 (14.3)	83 (56.8)	65 (44.5)	5 (3.4)
Wanting to solve problem on my own						
No	219 (55.7)	47 (21.5)	37 (16.9)	142 (64.8)	94 (42.9)	9 (4.1)
Yes	174 (44.3)	23 (13.2)	29 (16.7)	100 (57.5)	78 (44.8)	4 (2.3)

Abbreviations: ETB, Ethiopian Birr; WHODAS, World Health Organization Disability Assessment Schedule.

^a^ Student, jobless, or daily labor.

### Treatment Sources

Of the 393 participants experiencing symptoms, 66 (16.8%) contacted general health professionals, and 70 (17.8%) sought mental health professionals. In contrast, 242 (61.6%) turned to traditional healers, whereas 105 (26.7%) sought support from intimate partners (eTable in Supplement 1).

### Treatment Barriers

**Figure 1.**
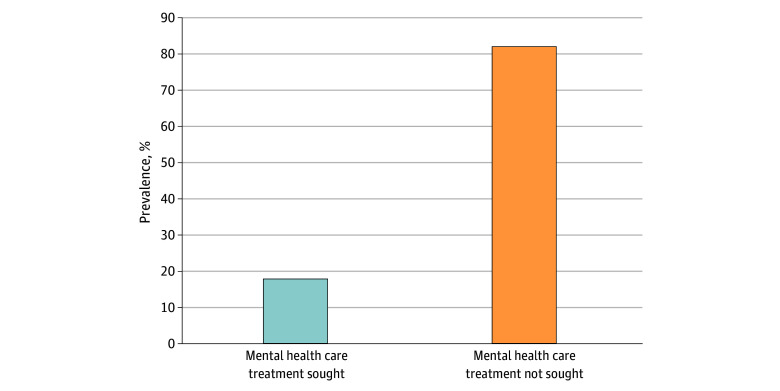
Prevalence of Treatment Gap Among Participants Using Holy Water With Mental Illness Symptoms, North Wollo Zone, Ethiopia, 2024

**Figure 2.**
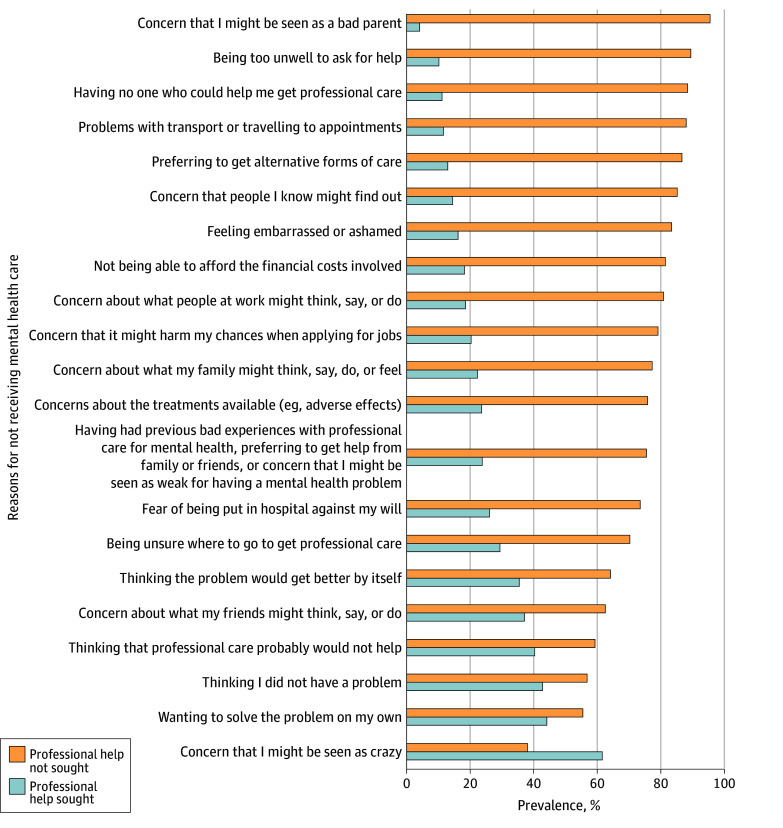
Reasons for Not Receiving Mental Health Care Among Participants Using Holy Water, North Wollo Zone, Ethiopia, 2024

Overall, 323 (82.2%) experienced a treatment gap, with 70 (17.8%) seeking professional help. A total of 221 (56.2%) did not seek support from family or friends (Figure 1). Participants identified key barriers to seeking professional help, including fear of being perceived as bad parents (377 [95.9%]), feeling too unwell to ask for help (353 [89.8%]), having no one who could help them get professional care (349 [88.8%]), and transportation issues (347 [88.3%]). Other barriers included concerns about being labeled “crazy” (150 [38.2%]), desire to handle the problem independently (219 [55.7%]), and believing there was no problem (224 [57.0%]) (Figure 2).

### Factors Associated With Treatment Gap

The analysis identified several factors associated with the treatment gap. Participants without a perceived need for treatment had higher odds of experiencing a treatment gap (AOR, 2.49; 95% CI, 1.09-5.71). Male participants had higher odds compared with female participants (AOR, 2.32; 95% CI, 1.20-4.48). Those with a college education or higher faced greater odds of a treatment gap (AOR, 4.34; 95% CI, 1.79-10.53). Low personal income was associated with higher odds of a treatment gap (AOR, 2.41, 95% CI, 1.72-4.14), and low family income also had higher odds (AOR, 2.56; 95% CI, 1.22-6.57) (Table 3). In addition, uncertainty about where to find help (AOR, 1.97; 95% CI, 1.21-4.53) and denial of mental health issues (AOR, 3.21; 95% CI, 1.24-6.43) were also associated with a treatment gap.

**Table 3.** Treatment Gap and Related Factors Among Participants With Symptoms of Mental Illnesses Who Sought Spiritual Healing Through Holy Water^a^

Factor	Treatment gap for mental health care, No. (%) (N = 393)^b^		Odds ratio (95% CI)		*P* value
	No	Yes	Crude	Adjusted	
Perceived need for treatment					
No	276 (70.2)	52 (13.2)	2.03 (1.09-3.77)^a^	2.49 (1.19-5.71)	.03
Yes	47 (12.0)	18 (4.6)	1.0 [Reference]	1.0 [Reference]	NA
Gender					
Male	147 (37.4)	45 (11.4)	2.16 (1.26-3.68)	2.32 (1.20-4.48)	.01
Female	176 (44.8)	25 (6.4)	1.0 [Reference]	1.0 [Reference]	NA
Residence					
Urban	94 (23.9)	29 (7.4)	1.0 [Reference]	1.0 [Reference]	NA
Rural	229 (58.3)	41(10.4)	0.58 (0.34-0.99)	0.56 (0.29-1.08)	.08
Marital status					
Married	118 (30.0)	14 (3.6)	1.0 [Reference]	1.0 [Reference]	NA
Single	104 (26.5)	32 (8.1)	2.59 (1.31-5.13	2.03 (0.83-4.97)	.12
Divorced	73 (18.6)	19 (4.8)	2.19 (1.04-4.64)	0.94 (0.35-2.51)	.89
Widowed	28 (7.1)	5 (1.2)	1.51 (0.50-4.53)	1.47 (0.39-5.57)	.57
Educational status					
Unable to read and write	162 (41.2)	18 (4.6)	1.0 [Reference]	1.0 [Reference]	NA
Primary level	63 (16.0)	15 (3.8)	2.14 (1.02-4.51)	2.31 (0.99-5.42)	.053
Secondary level	43 (10.9)	10 (2.5)	2.09 (0.90-4.86)	2.07 (0.76-5.63)	.16
College and above	55 (14.0)	27 (6.9)	4.42 (2.26-8.64)	4.34 (1.79-10.53)	<.001
Personal income					
Low	209 (53.2)	58 (14.8)	2.64 (1.36-5.11)	2.41 (1.72-4.14)	<.001
High	114 (29.0)	12 (3.0)	1.0 [Reference]	1.0 [Reference]	NA
Family income					
Low	217 (55.2)	60 (15.3)	2.93 (1.44-5.95)	2.56 (1.22-6.57)	<.001
High	106 (27.0)	10 (2.5)	1.0 [Reference]	1.0 [Reference]	NA
Unsure where to go					
No	208 (52.9)	33 (8.4)	1.0 [Reference]	1.0 [Reference]	NA
Yes	115 (29.3)	37 (9.4)	2.03 (1.20-3.42)	1.97 (1.21-4.53)	.01
Thinking has no problem					
No	215 (54.7)	31 (7.9)	1.0 [Reference]	1.0 [Reference]	NA
Yes	108 (27.5)	39 (9.9)	2.50 (1.48-4.24)	3.21 (1.24-6.43)	<.001
Professional help will not work					
No	184 (46.8)	50 (12.7)	1.0 [Reference]	1.0 [Reference]	NA
Yes	139 (35.4)	20 (5.0)	0.53 (0.30-0.93)	0.74 (0.25-2.23)	.59
Concern what friends think					
No	195 (49.6)	52 (13.2)	1.0 [Reference]	1.0 [Reference]	NA
Yes	128 (32.6)	18 (4.6)	0.53 (0.29-0.94)	0.87 (0.14-5.54)	.88
Wanting to solve problem on my own					
No	172 (43.8)	47 (11.9)	1.0 [Reference]	1.0 [Reference]	NA
Yes	151 (38.4)	23 (5.9)	0.56 (0.32-0.96)	0.97 (0.29-3.23)	.96
Internalized stigma					
Low	134 (34.1)	16 (4.1)	1.0 [Reference]	1.0 [Reference]	NA
High	189 (48.1)	54 (13.7)	2.39 (1.31-4.36)	2.76 (1.32-5.80)	<.001
Social support					
Poor	145 (37.0)	33 (8.4)	2.53 (1.12-5.73)	2.29 (0.87-6.00)	.09
Medium	89 (22.6)	29 (7.4)	3.63 (1.57-8.36)	2.89 (1.10-7.62)	.03
Strong	89 (22.6)	8 (2.0)	1.0 [Reference]	1.0 [Reference]	NA

Abbreviation: NA, not applicable.

^a^ This table shows bivariable and multivariable binary logistic regression analysis for the association between treatment gap and related factors among participants with symptoms of mental illnesses who sought spiritual healing through holy water in North Wollo Zone, Amhara, Ethiopia, 2024.

^b^ The care in this study includes only those participants who received the treatment from mental health professionals, including psychiatrists, mental health specialists, and psychologists.

### Qualitative Findings

Seven male KIIs and 3 FGDs with 19 participants (11 male and 8 female participants) were conducted. Data collection ended at thematic saturation. Interviews averaged 20 minutes, whereas FGDs lasted 45 minutes. Four key themes emerged from 26 participants aged 20 to 60 years.

#### Cultural Beliefs and Misconceptions in Mental Health Care

Many attribute mental illness to supernatural causes and first seek traditional healers; medical care is only pursued when these methods fail. “When they first become ill in rural areas, they go to a witch doctor and do not seek help from a medical doctor. If these remedies fail, they eventually come here (holy water).” (KII, St Abune G, Sanqa)

Some participants framed mental illness as a divine test beyond medical intervention: “Some people say this is the work of the Creator; there is no medicine for it.” (FGD2, St Giorgis, Gatira)

Misinformation about psychiatric medication and mental illness contributes to nonadherence. Many believe mental illness is spiritual and cannot be cured. One participant explained, “Many think mental illness is spiritual and that medication worsens the condition.” (FGD, St Urael) Another participant said, “There are many people in the community who are mentally ill, and this represents only half of those who have sought treatment. However, we don’t believe that they have fully engaged in treatment. Society’s perception is that, firstly, they do not think they can be treated or cured through psychiatric care, and secondly, due to superstition, they believe that the treatment will make them gain weight, hinder their ability to work, and prevent them from being active.” (KII, Woldia)

#### Socioeconomic Barriers to Mental Health Care

Participants with intermediate social support had 2.89 times higher odds of a treatment gap (AOR, 2.89; 95% CI, 1.10-7.62). Limited income and weak support hinder mental health care, leading many to spiritual remedies; caregivers face emotional and financial burdens, especially with aggressive patients.

“My son has been ill for seven years. I had to stop hospital treatment due to financial issues and took him to Wonkshet Gabriel Monastery, but there was no solution.” (FGD1, St Urael)

“I was begging to treat him now because I ran out of money, so I came here. I didn’t follow up on his medication because I don’t have a helper. In the past, I received help from two families. I was given a sum of one thousand birr, and with that money, I could go for treatment. When I didn’t get that money, I stopped the follow-up. If I had money, I could treat him, but there is no one to help me.” (FGD3, St Abune G, Sanqa)

“Though it is not well talked about in the media and in meetings about mental health, the society has little awareness and knowledge about mental health because the chance of getting it is low.” (KII, Woldia)

#### Stigma and Treatment Avoidance

Internalized stigma was associated with higher odds of a treatment gap (AOR, 2.76; 95% CI, 1.32-5.80). Stigma and shame isolate patients and families; fear and lack of support often lead to individuals being confined or restrained. “People are afraid of them [people with mental illnesses], what happened, people embarrass them, people don’t approach them, and social interaction decrements.” (KII, Kobo)

Families often hide their struggles due to fear of stigma as illustrated by the following 3 quotes: (1) “Patients or their families fear isolation and want to keep their illness private.” (KII, Woldia); (2) “They told me to tie him and hold him; no one can come close to me. (FGD2, St Giorgis Gatira); and (3) “People with schizophrenia come after 10 to 12 years, while others may seek help sooner.” (KII, Mersa)

Treatment is often stopped early due to stigma, financial issues, or misconceptions; some patients hide medication use after feeling better. “They stop treatment once they improve or fear being seen as weak.” (KII, Kobo)

#### Institutional Neglect and Insufficient Mental Health Services

Mental health care in Ethiopia suffers from underfunding, medication shortages, lack of inpatient care, and minimal institutional support, causing frustration among professionals. “Medication supply interruptions and lack of inpatient treatment are major challenges.” (KII, Mersa)

Participants recommended stable medication supplies, nongovernment organization collaboration, increased mental health awareness, and better health care worker support. “Hospitals must ensure a consistent supply of medication and educate communities about mental illness.” (KII, Woldia)

## Discussion

This study examined the mental health treatment gap among individuals seeking spiritual healing through holy water who exhibited mental health symptoms. The treatment gap was 82.2% (95% CI, 78.1%-86.0%),consistent with findings from India,^37^ Brazil,^38^ and a World Health Organization report.^9^ The higher gap observed may be attributed to the broader inclusion of all mental health conditions, unlike other studies focused on specific disorders, such as schizophrenia. Compared with countries such as Canada (17%)^39^ and Europe (50%),^40^ the gap was considerably larger, reflecting socioeconomic disparities and differences in health systems. The findings more closely resemble treatment gaps reported in Nigeria (46%)^41^ and Japan (67.7%).^42^

Key factors associated with the treatment gap included lack of perceived need for treatment, male gender, higher educational level, low personal and family income, uncertainty about where to seek help, denial of mental health issues, and internalized stigma. Specifically, participants with a college degree or higher had 4.34 times greater odds of experiencing a treatment gap (AOR, 4.34; 95% CI, 1.79-10.53). This finding aligns with studies from Germany,^43^ Singapore,^44^ and a systematic review^45^ suggesting that although a higher educational level increases mental health awareness, it does not always translate into help-seeking behavior, often due to persistent stigma and reliance on personal coping mechanisms.^45,46^

Not perceiving a need for treatment was associated with higher odds of a treatment gap by 2.49 times. Many, especially those in rural areas, prefer traditional healers and may not recognize mental illness as requiring medical care.^18^ Qualitative data emphasized the reliance on cultural practices, such as witch doctors and holy water, with medical care sought only when these traditional methods fail.

Male participants had 2.32 times higher odds of experiencing a treatment gap than female participants. This finding is consistent with global evidence indicating that women are more likely to seek mental health services, possibly due to social norms encouraging emotional expression,^47,48,49^ In contrast, masculine socialization, self-stigma, and coping strategies, such as substance use, discourage men from seeking help.^50,51,52,53,54,55^

Low personal income was associated with 2.41 times higher odds of a treatment gap and low family income with 2.56 times higher odds. Financial constraints, limited awareness, and stigma contribute to unmet mental health needs in low-income households, as supported by qualitative findings describing caregivers’ financial burdens and discontinuation of treatment,^47,56,57,58^ as supported by qualitative findings describing caregivers’ financial burdens and discontinuation of treatment

Uncertainty about where to find help increased the odds of a treatment gap by 1.97 times, whereas denial of mental health issues tripled the odds. Low mental health literacy prevents symptom recognition and timely help-seeking, whereas stigma and cultural beliefs lead individuals to favor traditional healing over psychiatric care.^17,59^ Raising awareness and normalizing mental health discussions can empower individuals to seek appropriate treatment. Qualitative findings support this.

Additionally, little concern was given to raising mental health awareness, resulting in a lack of awareness and not getting the appropriate treatment. Internalized stigma was associated with 2.76 times higher odds of a treatment gap. Shame and fear of discrimination cause avoidance of treatment and social isolation. This is corroborated by studies from Australia, the US, and Ethiopia and meta-analyses confirming stigma as a major barrier to care.^60,61,62,63^ The qualitative findings showed that

Similarly, another participant observed that patients and their families are often concerned about the stigma associated with mental illness. Additionally, a focus group participant shared a personal experience. Participants with intermediate social support had 2.89 times higher odds of a treatment gap, consistent with evidence that weak social networks and stigma hinder help-seeking.^62,64,65^ Strong social connections are essential for improving mental health service utilization^58,66,67^

### Strengths and Limitations

The study’s strengths include a mixed-methods design and validated tools. Limitations of this study included its cross-sectional nature, social desirability bias, selection bias due to the specific population studied, reliance on self-reported data, and lack of focus on specific disorders or service quality.

## Conclusions

In this cross-sectional study of individuals receiving holy water, we found an 82.2% mental health treatment gap, with only 1 in 6 individuals receiving care. This gap was largely attributed to cultural beliefs, stigma, and socioeconomic barriers. To address this disparity, we recommended the integration of traditional and biomedical health care systems, the implementation of community-based antistigma programs, and the strengthening of mental health policies to improve access to treatment.

## Notes

Supplement 1. **eTable.** Help Seeking Behavior Among Participants With Symptoms of Mental Illnesses Seeking Spiritual Healing Through Holy Water at Selected Sites in North Wollo Zone, Amhara, Ethiopia, 2024 (N=393) **eFigure.** Sampling Procedure for Selecting Holy Water Attendees, North Wollo Zone, 2024

Supplement 2.
Data Sharing Statement
