# Supplementary material for: Mental Health Treatment of Individuals Seeking Holy Water Treatment in Ethiopia
Source: JAMA Netw Open. 2025 Oct 8;8(10):e2536558. doi: 10.1001/jamanetworkopen.2025.36558 (PMC12508979; doi:10.1001/jamanetworkopen.2025.36558)
Supplement: Supplement 1. — eTable. Help Seeking Behavior Among Participants With Symptoms of Mental Illnesses Seeking Spiritual Healing Through Holy Water at Selected Sites in North Wollo Zone, Amhara, Ethiopia, 2024 (N=393) eFigure. Sampling Procedure for Selecting Holy Water Attendees, North Wollo Zone, 2024 [file jamanetwopen-e2536558-s001.pdf]

Supplemental Online Content

Demeke SM, Tegegne KM, Kidie AA, Denberu FG. Mental health treatment of individuals seeking holy water treatment in Ethiopia. *JAMA Netw Open*. 2025;8(10):e2536558. doi:10.1001/jamanetworkopen.2025.36558

eTable. Help Seeking Behavior Among Participants With Symptoms of Mental Illnesses Seeking Spiritual Healing Through Holy Water at Selected Sites in North Wollo Zone, Amhara, Ethiopia, 2024 (N=393)

eFigure. Sampling Procedure for Selecting Holy Water Attendees, North Wollo Zone, 2024

This supplemental material has been provided by the authors to give readers additional information about their work.

© 2025 Demeke S et al. *JAMA Network Open.*

eTable. Help Seeking Behavior Among Participants With Symptoms of Mental Illnesses Seeking Spiritual Healing Through Holy Water at Selected Sites in North Wollo Zone, Amhara, Ethiopia, 2024 (N=393)

Variables		Frequency (%)
Source of help		
Professionals		
Mental health professionals	No	323 (82.2)
	Yes	70 (17.8)
General health professionals		
	No	327 (83.2)
	Yes	66 (16.8)
Non professionals		
Friends		
	No	303 (77.1)
	Yes	90 (22.9)
Parents		
	No	311 (79.1)
	Yes	82 (20.9)
Relative/family member		
	No	375 (95.4)
	Yes	18 (4.6)
Intimate partner		
	No	288 (73.3)
	Yes	105 (26.7)
Phone helpline		
	No	389 (99.0)
	Yes	4 (1.0)
Traditional healers		
	No	151 (38.4)
	Yes	242 (61.6)
Another source		
	No	383 (97.5)
	Yes	10 (2.5)

© 2025 Demeke S et al. *JAMA Network Open.*

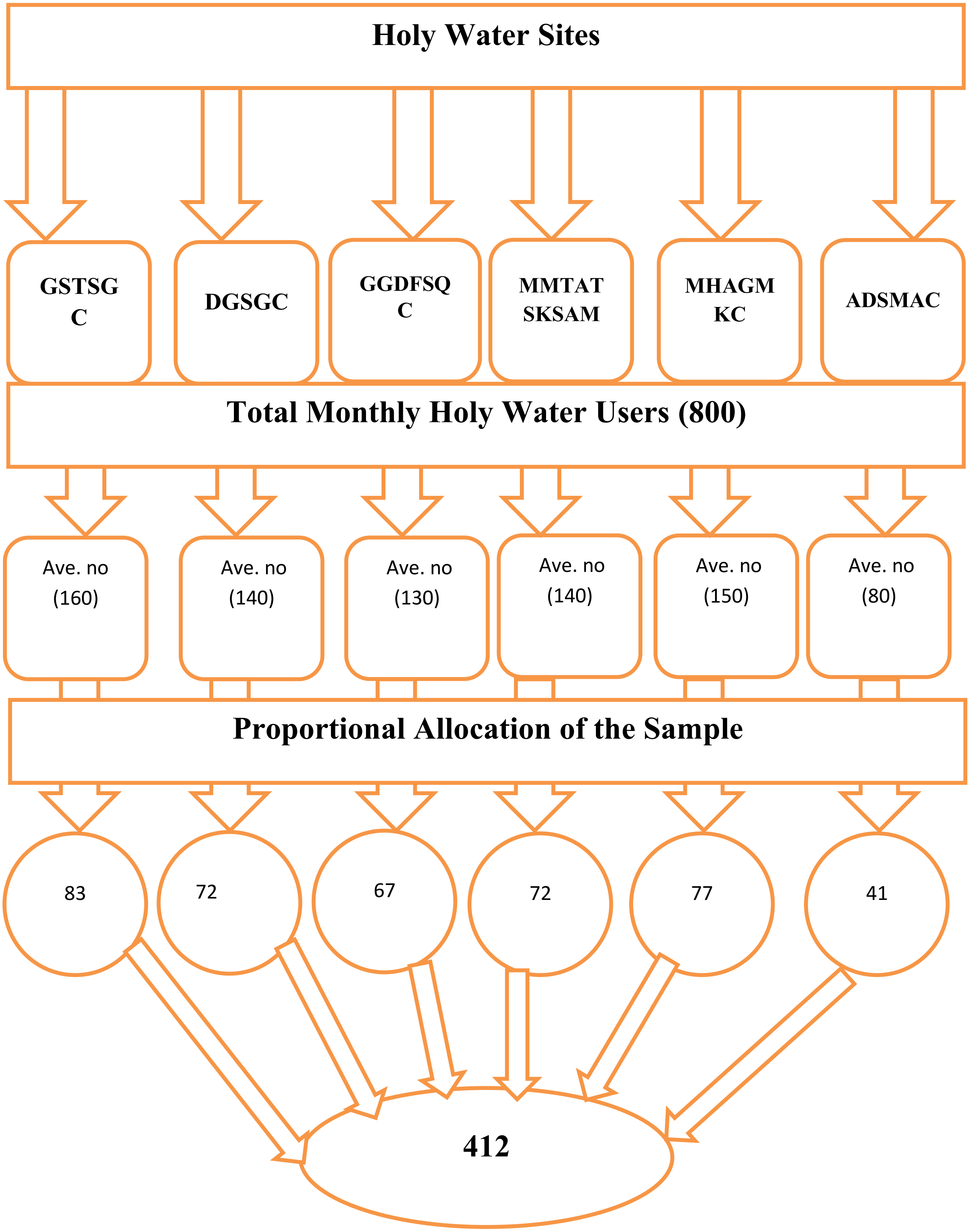

© 2025 Demeke S et al. *JAMA Network Open.*

eFigure. Sampling Procedure for Selecting Holy Water Attendees, North Wollo Zone, 2024

© 2025 Demeke S et al. *JAMA Network Open.*
