# Supplementary material for: Mental Health Treatment of Individuals Seeking Holy Water Treatment in Ethiopia
Source: JAMA Netw Open. 2025 Oct 8;8(10):e2536558. doi: 10.1001/jamanetworkopen.2025.36558 (PMC12508979; doi:10.1001/jamanetworkopen.2025.36558)
Supplement: Supplement 2. — Data Sharing Statement [file jamanetwopen-e2536558-s002.pdf]

Data Sharing Statement

Demeke. Mental Health Treatment of Individuals Seeking Holy Water Treatment. *JAMA Netw Open*. Published October 08, 2025. doi:10.1001/jamanetworkopen.2025.36558

Data
Data available: Yes
Data types: Deidentified participant data
How to access data: The datasets used and/or analyzed during the current study are available from the corresponding author on reasonable request.
When available: With publication

Supporting Documents
Document types: Statistical/analytic code
How to access documents: solmonmoge@gmail.com
When available: With publication

Additional Information
Who can access the data: Anyone requesting the data
Types of analyses: For a specified purpose
Mechanisms of data availability: With investigator support
